# Department of Selections

**Published:** 1857-06

**Authors:** 


### DEPARTMENT OF SELECTIONS.

*Reduction of a Femoral Dislocation, of Six Month's Standing, by Manipulation.* By Martial Dupierris, M. D., of Havana, Cuba.

Sir Astley Cooper, in his surgical work, asserts it to be imprudent to attempt the reduction of a shoulder dislocation after three months, and of the hip eight weeks, except in patients of very lax fibre or advanced age. But this great master admitted the existence of exceptions, and reported a case of dislocation of the head of the femur upon the dorsum ilii, reduced after the lapse of five years. The following case, although not very extraordinary, is instructive, as forming an exception to the rule laid down by authors of the highest standing.

The ship "Sword Fish," arrived at this port, on the 4th of June last, with a cargo consisting of three hundred and seventy-five Chinese colonists. Among the sick on board was a boy, about sixteen years old, called A-sin, who was taken to the infirmary. His skin was hot and dry; his tongue was parched; his pulse was very frequent and sometimes intermittent; he had a diarrhœa; and he lay upon his back with his thighs flexed upon the abdomen at a right angle, and his legs upon his thighs. He complained much of pain in the abdomen, and of excessive thirst. Our interpreter not understanding the patient's language very well, we deferred any further examination, hoping to procure shortly some one who could obtain from him a reliable history of the case. In the meantime treatment was ordered with a view to subdue the febrile action, and arrest the gastro-intestinal disorder.

After a month's careful attention, the fever was broken, but returned at irregular intervals in the form of short paroxysms, characterized by heat and perspiration; and the diarrhœa and intestinal irritation had entirely disappeared. Upon now being requested to get out of bed, he replied that it was impossible; and when he was lifted up by two nurses, he cried out with pain. I now discovered that when he was held in the erect posture, only the right foot touched the floor, the left being raised to the distance of seven centimetres; that the toe of the latter and the corresponding knee were inverted; and that his cries resulted from pain, produced by the nurse in an effort to make the left foot rest upon the floor. The peculiar deformity of the limb led me at once to suspect the existence of a coxo-femoral dislocation, and, upon further examination, my suspicions were confirmed by finding the head of the left femur upon the corresponding dorsum ilii.

Upon questioning the patient, through a new interpreter, in regard to the cause of the accident, I learned that before leaving China, while carrying two buckets of water suspended from the extremities of a pole placed across his shoulders, he slipped and fell upon his left side; that, being unable to rise, he was carried to the trading house, where he was treated by a native physician, and, after several days, was able to walk with a stick, without being able, however, to get his foot to the ground. After going on board the vessel, his sufferings increased, and he was obliged to keep upon his back during the entire voyage. In the meantime, he lost his appetite, and his bowels became deranged.

I have already described the condition of the patient upon his arrival in this country. It only remains to state, that by the time I had succeeded in arresting his fever and diarrhœa, he was extremely emaciated, and his muscular system very much enfeebled.

It was now the early part of July, and I determined, after a careful consideration of the case, to attempt the reduction; at the same time, I was fully awaie that, in order to compel the head of the bone to abandon its new position, to break up the adhesions which held it fast, and, finally, to replace it in its origiual position, it was necessary to employ an amount of force, which seemed hardly justifiable in a constitution so debilitated. I appreciated the danger, and also the necessity for action; for the constant pain, augmented by the least movement, would have undoubtedly brought on a marasmus, terminating sooner or later in death.

The permanent contraction of the surrounding muscles, almost universally present under similar circumstances, was wanting in this case; as before stated, they were all in a flaccid condition, except the great gluteal, which was painful to the touch. The limb was somewhat more emaciated than its fellow of the opposite side, owing doubtless to the obstruction to the circulation and enervation, produced by adhesions of the vessels and nerves in their unnatural position.

I did not think it prudent to submit the patient to the action of chloroform or ether, owing to his feeble condition, but was obliged to operate without the aid of these valuable means. The following method was pursued:

The body being held by two assistants by means of two bands, one of which passed beneath the perenium, and the other under the axillæ, traction was made upon the limb by two strong and intelligent assistants. The movement of the head of the bone, resulting from this manœuvre, was very limited, even when the force was much increased; and the excrutiating pain, which the patient referred to the iliac region, compelled us for the moment to desist.

The following day, the patient having obtained a tolerable night's rest by means of a narcotic potion, I concluded to attempt the reduction by flexion, believing that I could thus better prevent any accident which the necessary force might produce; the operator, in adopting this method, having it in his power to follow the head of the bone by pressure upon it with the hand, aiding its movement in the proper direction, or correcting any deviation that may occur. The emaciated condition of the boy was eminently favorable for such a procedure.

The patient being placed upon his back, and the trunk of the body made steady by assistants, with the left hand I grasped the upper part of the leg, placed the right hand upon the head of the bone in the iliac fossa, and then proceeded to flex the leg upon the thigh, and the thigh upon the pelvis. By this movement the great gluteal muscle was relaxed, and the head of the bone advanced, while with the right hand I directed the latter toward the cotyloid cavity. As soon as I judged the head to be immediately above the centre of the socket, I extended the leg, the thigh remaining flexed at a right angle; and then using the limb as a lever, I rotated it from within outward, and at the same time extended it by making a movement of circumduction in a similar direction. When by these procedures the limb was brought near to its opposite fellow, a snap, audible to the assistants, and of a deeper character than is ordinarily observed in the reduction of recent dislocations, indicated the return of the head of the bone to its natural position; a fact which was farther substantiated by the establishment of the original length and form of the member and the subsidence of the pain.

The after-treatment consisted in placing a pad between the knees, and another between the internal malleoli, and confining the limbs together by two bands, one above the knees, and the other around the lower part of the legs. But in spite of these precautions to prevent re-displacement, the next morning I found that the dislocation had been reproduced. It was again reduced, but for three successive days there was a re-displacement. After this, however, the head of the bone kept its place; passive motion was daily employed, and all suffering ceased. After twenty days of rest, and a liberal use of the lactate of iron, the patient was allowed to get up; and, being provided with a pair of crutches, upon which he exercised himself daily, improved very rapidly. The muscles gradually recovered their bulk and vigor; and at the end of forty-eight days he was enabled to walk without crutches, although with some fear of falling. About the middle of August, he was put to work in a cigar manufactory, and has continued well ever since.

*Remarks.*—Sir Astley Cooper remarks, that a surgeon should carefully consider before deciding to attempt a reduction of an old dislocation, especially in a young man. As a general rule, the following conditions are recognized as favorable to the operaton. First, that the head of the bone is movable, as may be ascertined by making direct pressure upon it, or by moving the limb in various directions; secondly, that the situation of the head is such as to admit of subcutaneous incisions of the adhesions, if this should become necessary; thirdly, that the acetabulum is proved to be empty; fourthly, that the limb is shorter than its fellow; and, lastly, that the patient's general health is good. In the absence of any of these conditions, surgical authorities advise us against any violent attempt at reduction. In the case above reported, it was impossible to prove, whether or not, the cavity of the acetabulum was filled up, although I made the most careful examination; and as to the mobility of the head of the bone, the intolerable pain which accompanied the manipulations, enabled me to obtain this only to a very limited degree; moreover, the patient's constitution was in a very debilitated condition. Notwithstanding, however, these adverse circumstances,—circumstances which, we are advised to consider as forbidding any attempt at reducion,—I felt compelled to act, resting my judgment upon the fact, that the principal cause of the patient's rapidly failing health was the displacement of the bone. The result of the case proved the correctness of my opinion.

In reference to such cases, I have always adopted, as a diagnostic rule, that, so long as acute pain continues to be felt during movements of the limb, the great probability is, that the work of new formation is not complete, and, therefore, the cavity of the ocetabulum not entirely obliterated. This circumstance was present in the foregoing case, and, as I considered the cause of the patient's debilitated condition, it was my duty to remove the cause if possible. Besides, it was possible, indeed likely, that the enfeebled state of the system had delayed the formation of adhesions and a new cavity, and the contraction and transformation of the surrounding muscles. In proof of the latter, I need only refer again to the statement already made, that all the surrounding muscles, except the great gluteal, and, possibly, the triceps, were flaccid, and devoid of contractility; while, on the contrary, in the iliac region there was acute pain, with increased heat, discoloration, and tension of the skin, indicating the presence of inflammation, which sufficiently accounted for the fever sometimes observed. And it is reasonable to suppose, that, if the patient had not observed perfect rest, the increased pain would have given rise to more frequent exhibitions of fever. Thus, having discovered that the only point at which inflammation existed, corresponded to the situation of the head of the femur, it was natural to suppose, that the close contraction of the great gluteal muscle upon this foreign body was due to an effort of nature to get aid of the latter. The indication, therefore, seemed to be to aid, rather than to supplant the natural powers ; but the contraction of the muscle upon the neck of the bone had produced an opposite effect, and it was necessary, therefore, to relax it, in order to remove the head from its abnormal position. For this purpose, I resorted to flexion of the thigh, seeing that traction upon the limb only increased the difficulty.

It is generally believed that no noise accompanies the reduction of an old dislocation, but in the foregoing case the sound was deeper than usual, and loud enough to be heard by all the assistants.

As soon as the great gluteal muscle was relaxed, the pain was much diminished ; and when the bone entered the socket, the patient exclaimed that he was cured, and expressed a desire to get up immediately. But notwithstanding the relief thus suddenly obtained, the cure was by no means complete, for the muscles and ligaments, torn by the dislocation, had not the power to retain the head of the bone in its place, and hence the necessity for subsequent quietude of the limb. But with an impatient boy, tired of a six months' confinement to bed, this was no easy matter ; and after several re-displacements and reductions, I determined to let him suffer awhile, in order to impress upon his mind more forcibly the necessity of keeping quiet. The hope of accomplishing a perfect cure was, however, much increased by the greater ease with which each successive reduction was performed. Indeed, the last time that I had occasion to perform the operation, the reduction was accomplished by simply pushing the head of the bone back into the socket.

In conclusion, I may be permitted to state, that the period within which an old dislocation may be reduced cannot be fixed *a priori* ; for some cases, which cannot be remedied by one method, may perhaps be by another. I would say that, so long as there is decided pain in the situation of the displaced bone, it is justifiable to attempt reduction ; for the existence of pain indicates that the process of filling up the natural cavity, and the formation of a new articular surface are not complete. The objection may be urged that the natural cavity is no longer surrounded with ligaments capable of retaining the bone in its original position, but the same may be said of the new; and nature will certainly restore the necessary attachments sooner in a natural, than create them anew in an unnatural situation.—*N. A. Medico-Chir. Review.*

---

*On Nux Vomica in Constipation.* By J. H. Houghton, Esq. Dudley.

[We are constantly consulted by patients who tell us that they hardly ever have their bowels moved without taking medicine. They have generally tried every kind which we can recommend, and the only consolation we can give them is, that they must ring the changes and increase the doses. The effect of this is often only to aggravate the mischief and hasten on some of the thousand and one ill consequences which we may expect from such a state of affairs. As the result of much experience, Mr. Houghton says that in nux vomica we have a remedy capable of relieving many cases of this nature, of which he gives the following :—]

Case 1. December 4th. Emma Gibbs, aged 29, came under my care at the Dispensary on October 3d, suffering from an attack of congestion of the uterus and vagina, which yielded to local depletion, rest, baths, &c. She is naturally of a delicate frame and constitution, and was left very much debilitated by the attack. She got relief to her debility by taking quinine and iron; but during the whole of the time her bowels were unmanageable and obstinately costive. To relieve this, she has taken, and had given to her, castor oil, senna mixture, pills, and, last of all, pills containing two parts of colocynth and one of henbane. Of these, at first, she took two with relief; then three became necessary, and then four; she then took four at night, and followed it by castor oil in the morning, and thus obtained a motion once in two or three days, with much pain and trouble. On the 13th of November I gave her twelve pills, consisting of dr. ss of of henbane, sc j. of compound extract of colocynth, and gr. iij. of extract of nux vomica, and desired her to take one every night, and to continue her tonics as usual. From that time to the present (three weeks,) she has taken one pill every night, and had one comfortable motion every morning, without the aid of any other aperient, and her health has much improved.

Case 2. Sarah Silvester, aged 35, applied to the Dispensary on December 16th, suffering from a severe attack of gastrodynia, attended by some derangement of the uterus. I extract the following from my notes:—Tongue furred, yellow, indented by the teeth, moist,

Appetite bad ; violent pain after eating, worse at times ; frequent regurgitation of food, sometimes vomiting ; sometimes she is compelled to produce vomiting before she can get relief after eating. Bowels habitually costive, and very unmanageable. Her habit is to take medicine twice a week, after which she has two or three stools, and then the bowels do not act again till she again takes medicine. She says she has taken "all sorts of medicine," including many quack pills, for the relief of her bowels, but only with temporary benefit, the bowels returning to their inactive state. She had bismuth three times a day, and the pill before named every night.

December 19th. One motion daily, with perfect comfort ; she has not been so comfortable in her bowels for years. Gastrodynia and vomiting much relieved.

January 16th, (thirty-two days). She takes one pill every night, and had one motion every day with comfort The pills have never missed. Her stomach symptoms are relieved.

February 13th. She was at the Dispensary to-day. She has taken one pill every night, now two months, and it has never failed.

Authorities are very silent on the peculiar property of the nux vomica which I am now discussing. The last edition of "The Pharmacopœia Londinensis" dismisses the whole matter in these laconic words: "Use—in some cases of paralysis."

Pereira does not allude to it, though he speaks of the efficacy of the drug in "dyspepsia, pyrosis, and some form of dysentery."

Dr. Copland, whose mind seems to have embraced almost every thing in medical science, says, "In cases apparently depending on deficient tone of the muscular coat of the large bowels, and imperfect propelling power of the upper part of the rectum, I have seen benefit from combining the extract of nux vomica with the pilula aloes c. myrrha, or compound extract of colocynth."

Dr. Neligan, in his excellent treatise "On the Uses and Modes of Administration of Medicines," observes: "I have used the extract of nux vomica with much advantage, as an addition to purgatives in constipation depending on want of tone in the muscular coat of the large intestines, one of the most frequent causes of this state in females, and one which is distinctly characterised by great secretion of flatus and colicy pains which accompany it."

So far as I have been able to learn, we are indebted to Magendie for the first suggestions on the powers of nux vomica. In 1845, Dr. Tessier, of Lyons, published a paper which was quoted in "The Lancet," and in which he says that "he considers it particularly indicated in cases where there is reason to suspect general want of tone in the bowels, as in paralytic and old persons, or where we suspect want of tone of the muscular coat, in consequence of great and long-continued distention; or, in short, where the constipation can be referred to an undue secretion of gas, which in itself, by causing distention of the bowels, diminishes their contractile power."

In the Journal for this association for May, 1848, is an article by Mr. Boult, of Bath, on the employment of nux vomica in habitual constipation, in which he observes: I first tried the extract alone, in half grain doses, two or three times a day, and was disappointed with the result. I was then led to use the extract in combination with aloes, rhubarb and scammony, and was surprised at the result." Mr. Boult seems to think *that it has the power of increasing* the action of other purgatives; and he says: "Generally speaking, a pill containing three-quarters of a grain of Barbadoes aloes, three-quarters of a grain of extract of rhubarb, and half a grain extract of nux vomica, taken at bed time, will produce one or two evacutions the next morning." And he continues: "I have prescribed the pill already mentioned for months together, and at the end of that time the effect has been produced as certainly as at first, and no bad consequence has arisen; on the contrary, I think it will be found that, when the medicine is discontinued, the tendency to costiveness will be found to be diminished."

The correspondent of The Medical Gazette, November 10th, 1855, in his notes on Hospital Therapeutics, has the following admirable remarks on the subject: "Among the conditions over which nux vomica, and its active principle, strychnia, possess most useful powers, is that of habitual constipation from muscular atony of the intestinal tube. At the City Hospital for Diseases of the Chest, we observe that Dr. Peacock and Dr. Andrew Clark are both in the habit of frequently resorting to it for this purpose. It is generally given in combination with the compound rhubarb pill, and in doses of the extract of from one-sixth to one-half a grain. Of itself it can scarcely be deemed an aperient; that is, it does not so much excite peristaltic action as supply tone to the weakened muscular coat, by which it is enabled to reply efficiently to other irritants. Hence the need of combination with aloes, rhubarb, or some similar drug."

Dr. Peacock has mentioned to us a case in which a man of feeble intellect and torpid nervous system generally, had derived great benefit from its employment. At first, the bowels were obstinately costive, and lavements produced no action: but since the use of nux vomica they have so far increased in power and susceptibility that simple injections are quite sufficient to procure all the action that is necessary.

With the observations quoted I generally concur, but especially with those of Mr. Boult and the correspondent of the Medical Gazette.

From the facts and opinions adduced I think we may safely infer—

1. That in the nux vomica we have a new agent in the treatment of constipation; not a purgative or aperient, but a substance which, added to very minute doses of various purgatives and aperients, forms a kind of *tertium quid*, which combines the advantages of purgatives without the disadvantages, which does not leave the bowels debilitated and indisposed to act after its operation, but which, on the contrary, imparts tone, rendering their action more certain.

2. That the agent does not lose its power by contiued use.

3. That it is a perfectly safe remedy when used in the mode suggested.

4. That it is not an accumulative medicine.—*Assoc. Med. Jour.*

---

*Influence of the Obliteratiou of the Vena Porta on the Secretion of Bile, and on the Glycogenic Function of the Liver*, *L' Union Medicale*, Sept. 14, 1855. Translated and condensed from the French: By M. Morton Dowler, M. D.

M. Ore addreesed a memoir under this title, in which he reports a series of experiments made with a view of studying the influence of the obliteration of the vena porta on the secretion of bile. These experiments have been undertaken by request of M. Gintrac, (of Bordeaux,) with reference to a case, occurring under the observation of this physician, which seemed to weaken the physiological theory which contemplates the vena porta as furnishing the liver the materials of the biliary secretion.

In the first two series of experiments, the vena porta was tied or obliterated by different processes, and it was found that in spite of this obliteration, the gall bladder was constantly full of bile, and that the matter contained in the intestine was colored by this secretion.

In a third series of experiments the author investigated, at one and the same time, the influence of the obliteration of the vena porta on the biliary secretion, and one on the glycogenic function of the liver. The four experiments of these series, appeared to him to confirm, in the most positive manner, the theory of the formation of sugar in the liver, established by M. Claude Bernard.

This physiologist, says M. Ore, has seen the secretion of sugar disappear from the liver when the organ has become diseased. This fact finds confirmation in one of the experiments of the third series, in which the liver, presenting abscesses in one of its lobes, did not contain sugar in this part, but on the other hand, contained abundance of it in the portion of the organ which retained its normal condition.

In another experiment, in the same series, the liver presented abscesses throughout its whole extent, and did not contain the least trace of sugar, which is a new confirmation of what we have before said.

And, finally, in the last experiment, he has found abundance of sugar in the liver, though the vena porta was obliterated, and the substances resulting from the transformation of alimentary materials, that is to say, albuminose and glycose, could not any longer penetrate into the organ. This last experiment shows clearly, that the liver, as a reservoir of sugar, does not receive its supply, as such, by alimentation.

The author sums up his memoir by drawing the following conclusions :

1. The secretion of bile, having continued notwithstanding the partial or complete obliteration of the trunk of the vena porta, I hence conclude that it is not the blood of this vein which furnishes the materials of this secretion. It is at the expense of the blood of the hepatic artery, that the liver secretes this fluid. The biliary, like all other secretions, is therefore effected at the expense of the arterial blood. I have shown in my memoir why the obliterations of the hepatic artery cannot serve to determine the question, and how these obliterations show nothing tending to invalidate the conclusion which I have announced.

2. The secretion of sugar, by the liver, not having been changed by obliteration of the portal vein, it becomes evident that the production of sugared matter, as has been showu by M. Claude Bernard, results from the action of the liver itself, independently of alimentation.

3. The materials—albuminose and glycose—resulting from the digestion of amylaceous and albuminoid matter, not being able to traverse the liver, are not however lost to the organism, as may be realized by the anastomotic circulation existing between the superior mesenteric vein and the vena cava inferior.

4. And lastly, it is with the greatest reserve I would put forth the conclusion in the form of the question ; may not the arterial blood play a similar part in the formation of the hepatic sugar, that it does in the formation of bile ?

M. Andral, who was present, cited a fact that he had observed, in the course of his practice, a case which afforded results in perfect accordance with those obtained by M. Ore in the experiments above reported. A patient whose morbid manifestations led him to suspect the existence of obliteration of the vena porta, (an obliteration which in reality existed in the most absolute manner, as was verified by the autopsy,) not only did not present any symptoms indicative of a suspension of the biliary secretion, but furnished the proof that the glycogenic function still persited, for this patient was diabetic.—*N. O. Med. and Sur. Journal.*

---

*Mercurial Muco-Enteritis.* By Wm. H. Byford, M. D., of Evansville, Indiana.

If I were asked what was the most constant evidence of the constitutional effect of mercury, I should say inflammation of the mucous membrane of some portion of the alimentary tube. Doubtless, the most frequent site of this specific inflammation is the mouth, but certainly not always, or exclusively. In a vast number of cases in which mercury is administered with a view to its general effect, we are annoyed, if not interrupted in its use by excssive irritation of the bowels, before the gums are "touched," or a metallic taste is experienced by the patient. This irritation is, I think, commonly regarded as an immediate cathartic impression, or the purgative effect of super-secretion from the liver. Careful and somewhat extensive observation induces me to believe that both of these last are the effects of a specific mucous inflammation, just as salivation and enlargement of the salivary glands are produced by the inflammation of the mucous membrane of the mouth. Every body is familiar with inflammation of the mouth and salivation from mercurial inunction. When the mercury is taken internally, or applied externally, it will produce stomatitis.

This is a rule. I am satisfied that it will also produce duodenitis, colonitis, and rectitis, and when persisted in too long, these may become unpleasant, if not dangerous complications. We should always therefore, regard copious serous stools, with burning pain in the epigastrium and umbilical regions, or tenesmus and frequent mucous and bloody stools, as an indication for the withdrawal of the remedy, as having produced its specific effect upon the organism. These reflections have resulted from observing cases like the following:

In March, 1855, while treating V. R., a remarkably stout young man, of bilio-sanguine temperament, for traumatic iritis, I gave him calomel and opium for three or four days at inteavals of three hours. At the end of the fourth day, on visiting R. in the evening, I found him with excessive tenesmus, burning pain in the rectum, constant disposition to stool, &c. In fact, the symptoms were thoroughly dysenteric. The discharges were small, and consisted of mucous and blood, colored with bile. He stated that about 10 o'clock in the morning he had two large bilious evacuations, succeeded by these small ones; that he had to be up every ten minutes, and felt like it would be a relief to discharge the contents of the whole abdomen. In a few days, by the use of large opiates, cold injections, mucilaginous drinks, and fomentation to hypogastrium, he was entirely relieved from the symptoms. As the inflammation of the iris, although better, was not subdued, I thought it advisable to use more mercury, and to avoid, as I hoped I could, the irritating effects upon the bowels, I resorted to inunction. In about a week's continuance of it externally, the same sort of an attack supervened, and produced so much prostration that I was uneasy as to its results. He, however, recovered from it in a few days. In both instances there was no stomatitis whatever, and no increased flow of saliva.

M. W., about forty years of age, came to me laboring under a severe form of chronic opthalmia, attended with extensive ulceration of the cornea. After proper depletion—for he was robust and plethoric, I tried to indnce mercurialization as soon as possible. In four or five days, he was attacked with gripings, abdominal fulness, burning pain in the epigastric and umbilical regions, followed by copious serous discharges for three or four evacuations in quick succession. The discharges became gradually less, and in the course of seven or eight hours were very small, and composed of bloody mucous, attended with tenesmus, and excessively frequent attempts to evacuate, and constant desire to do so. These symptoms required active treatment for two or three days before they were subdued. Twice after this the same patient was brought under the influence of mercury, once by internal administration, and once by inunction. In both instances, the same symptoms were present.

I have selected the above two cases to show the more severe form of intestinal inflammation caused by mercury, and also because they were both apparently in good health and sound in every respect, except the eyes, I have had many similar cases in my practice since my attention has been thus directed, and I find the same symptoms to result indifferently in the cases of inunction or administration by the mouth. It is not usual for the symptoms to be so violent as in the cases I have narrated. More frequently the symptoms form more gradually, and do not attain this severity.

Although neither of these cases showed any signs of ptylism, others that occurred were complicated by this symptom. These cases have so often occurred in my practice that, although it would be unnecessary to describe them, I have been led to ascribe especial importance to them, and regard them as presenting one of the specific effects of mercury. The importance of attending to these manifestations will be apparent, when we reflect on the effect of mistaking them for adventitious occurrences, or its mere purgative influence. A continuance of the remedy guarded by opiates, after these unequivocal signs of saturation, would be to aggravate them to a dangerous degree.

Last year I was called, in council with another practitioner, in a case of pneumonia, in which the patient, as I thought, died from the additional prostration caused by mercury, in hypersecretion and exhausting pain and efforts at stool. The patient was about fifty years of age, rugged, and in so good a state of health as to warrant pretty copious depletion, tart. antimony and mercury as very appropriate treatment. On the eighth day, the intestinal irritation above described supervened, and from its obstinate continuance, on the fourth day after its first appearance, the patient died. I inferred that the fatal result was brought about by the secondary disease arising from mercury, from the analagous cases I had before observed, and the fact of the pulmonary symptoms having subsided two days before death.

These are instances of the cumulative and explosive efiects of mercury, and must not be supposed frequently to occur; and, as will readily suggest itself to the thinking physician, are parallels in this respect to the destructive sloughing stomatitis, which may either kill the patient or mutilate him for life. With proper circumspection, neither of these need be more than exceedingly rare accidents. I address the profession in this way in order to express my conviction that the inflammation in different portions of the alimentary tube accompanying the use of mercury is, equally with ptyalism, a sign of mercurialization, and indicating the same necessity of withdrawing, either partially or entirely, the cause which produces it, instead of combining opium with it to guard the bowels. This last course is like smothering a fire with an increase of combustible materials.

It may be said that the cases in which intestinal inflammation is manifested occur only in persons of peculiar or idiosyncratic organization, However this may be, I am sure that it is not a rare phenomenon. I would remark that where mercury produces a mild sore mouth or laudable salivation, we may expect its beneficial effects upon disease to be greatest. On the other hand, when it is attended with much sloughing of the gums, cheeks or lips, its good effects are not so likely to follow, This is the rule, I think also, as to the comparative intensity and its good constitutional effects in intestinal inflammation.

It is impossible I think, to predict from the appearance of a patient, what will be his susceptibility in this respect; but I have found intestinal irritation to occur more frequently in persons who were difficult to affect by it, and require several days to influence them with it.

I do not think these facts should deter us from using mercury in any case where it is indicated. They should merely inculcate great care and watchfulness in administering it, and the necessity of an intelligent understanding of all its earlier manifestations. In this way we may circumscribe its power and control it for good. I confess that I am one of the few, or many, whichever it may be, who do not believe that mercury will ever be entirely replaced by a substitute. Every year, it is to be hoped, will teach us better how to use it; but I do not think we will ever learn to do without it. One fact should always be remembered, and that is, that it is a powerfully exhausting remedy, and the freedom or cautiousness in using it must depend upon the stage of the disease and the vigor of the constitution. The early stages of sthenic inflammation is the time to use it most freely; and as the disease advances into the stage of structural lesion, much more caution is required to keep from doing harm. Always, when we have time, as in chronic or sub-acute disease, its good effects will be greatly enhanced by its very slow administration. In chronic cases, it will very advisable to spend two or three weeks in bringing the system under the influence of mercury.

The conclusions to which I have arrived from considerable experience, and which I think are derivable from this paper, are, that the specific acute inflammation produced by mercury has its site, 1st, most frequently in the mouth; 2d, very frequently in the lower portion of the colon and rectum, 3d, not so often in the dudenum; 4th, situated in all these localities, it may be combined with stomatitis or not; 5th, that inflammation may be produced in any ot these localities either by internal use or by inunction, and probably also by fumigation.—*Amer. Jour. Med. Science.*

---

*On the Vapor of Amylene.*—Dr. Snow read before the Medical Society of London, (Jan. 10, 1857,) a paper on this subject.

He said that amylene was first discovered and described in 1844, by M. Balard, Professor of Chemistry at the Faculty of Science of Paris. It was made by distilling fusel oil with chloride of zinc. M. Auguste Cahours had given the name of amylene five years previously to a product which was isomeric with it, and was made nearly in the same manner, but was now termed paramylene. Amylene itself was a colorless and very mobile liquid of extremely low specific gravity. M. Balard had not stated the specific gravity; but he, (Dr. Snow,) had found it to be 0.659 at 56 deg. It was very volatile, boiling at 102 deg. Fahr., and the specific gravity of its vapor was 2.45. It was a compound of ten atoms of carbon and ten hydrogen, and it bore the same relation to fusel oil, or alymic alcohol, that olefiant gas, or ethylene, bore to common alcohol. It burnt with a brilliant white flame. It was soluble in alcohol and ether in all proportions, but was very sparingly soluble in water. As far as he could ascertain, it required rather more than 10,000 parts of water for its solution. It had an odor somewhat resembling naphtha, some persons thought the odor agreeable, and some thought it unpleasant; the odor was not so strong or permanent as that of sulphuric ether, and it did not remain long in the patient's breath. The vapor of amylene was much less pungent than those of ether and chloroform, and, therefore, it was much easier to breathe, and had not caused coughing, except a little in two patients with catarrh. He was not aware of the existence of amylene till a few months ago, or he would have tried it sooner; for' judging from experiments which he had made on analagous substances, there could be no doubt of its causing insensibility when inhaled; but he could not tell, without trial, whether it might not be too powerful, otherwise objectionable, in its action. He made several experiments on small animals with amylene, and after inhaling small quantities of it himself, he administered it in King's College Hospital, commencing with cases of tooth drawing, on Nov. 10th, 1856, and he had more recently given it in the larger surgical operations. He found from experiments on animals, that to induce a very complete state of coma, which he called the fourth degree of narcotism, it required that a fifth part as much amylene should be absorbed as the blood was capable of dissolving. To cause the second degree, or that state in which consciousness and volition were disordered, but not abolished, it required a tenth part as much as the blood would dissolve; whilst to induce the third degree of narcotism, which was as far as he had found it necessary to carry the effect in the human subject, it required an intermediate quantity, or about fifteen per cent. In the case of chloroform, ether, and several allied substances, the proportion which required to be absorbed was far less, being only, for the fourth degree of narcotism, about one twenty-eighth part as much as the blood was capable of dissolving. Benzin, which was a simple carbo-hydrogen, like amylene, was intermediate between this and the above substance in the relative amount of it which was absorbed, one-seventeenth part as much as the blood would dissolve being required to induce the fourth degree of narcotism. Whilst the relative amount of amylene absorbed was high, the actual amount was extremely small, owing to its very sparing solubility in the serum of the blood and other watery fluids. He calculated that in the adult human subject the amount of amylene circulating in the system, in the third degree of narcotism, was less than three minims. Viewed in the light of the small quantity which required to be absorbed to cause insensibility, amylene was a very powerful agent; but when considered in relation to the quantity which was consumed during inhalation, in the usual way, it was very far from being powerful. This arose from the great tension and the small solubility of the vapor, in consequence of which it was, with the exception of a small fraction, expelled from the lung, again without being absorbed. It took from three to four drachms of amylene to cause insensibility in the adult, whilst less than a drachm of chloroform was usually sufficient. The quantity of sulphuric ether required to cause insensibility in the adult was eight to ten fluid-drachms, one-half of which was absorbed into the blood. In a protracted operation, the quantity of amylene used was greater than that of sulphuric ether, as the small quantity of the former which was absorbed was quickly exhaled again from the lungs, and required to be constantly replaced, whilst the large amount of sulphuric ether, when once absorbed, took a much longer time to evaporate in the breath. It was necessary for the patient to breathe air containing not less than fifteen per cent. of vapor of amylene, in order to reach the third degree of narcotism, or that condition in which consciousness and voluntary motion are entirely suspended, the pupils being usually contracted and turned upwards, but the muscular system not necessarily relaxed. The patient must inhale the amylene at the rate of rather more than a fluid-drachm a minute; in this way he becomes insensible in three minutes, or rather less; but if the vapor was not inhaled in a sufficient volume, he would not become insensible by continuing the inhlation, for however long a time; the quantity of vapor must be increased, or it would not succeed. He had administered the amylene in his ordinary chloroform inhaler, which he had, however, got somewhat enlarged. In the use of amylene, absence of pain had been obtained with less profound coma than usually accompanied the employment of chloroform and ether. There were some cases, indeed, in which the minor parts of an operation, under these latter agents, might be performed without pain, whilst the patient was in a semi-conscious state, or even altogether conscious; but they formed the exception; whilst in the use of amylene, the patient had very often been partially conscious during the operation. In a case that day, in which Mr. Fergusson removed a large melanotic tumour from the groin, the man repeated some verses very accurately whilst the arteries were tied, and was awake and talking to the bystanders whilst the wound was being stitched up, but felt nothing of it. The pulse was increased in frequency and force during the inhalation of amylene, to a greater extent than happened with chloroform; the respiration also was very often accelerated, about as often as in the inhalation of ether, and more frequently than with chloroform. There had not been much increase of saliva from the use of amylene, and he (Dr. Snow,) had not yet met with the profuse flow of saliva which was often troublesome in the employment of chloroform and ether. There had been no sickness in any of the 21 operations in which he had exhibited the amylene, nor any of the depression which so often preceded and accompanied the sickness from chloroform and ether; and there had been hardly any struggling or rigidity in any of the patients, although several of them being robust men, a good deal of both might have been expected before complete insensibility, if chloroform had been the agent employed. He was of opinion that amylene would be perfectly safe with careful management. Sulphuric ether seemed perfectly safe in whatever way it was used; although it had been blamed for causing death, no fatal accident seemed to have been really occasioned by it. This arose from the circumstance that the dose of ether occupied so much space in the form of vapor, that it could not enter the system except by degrees, and its effects were necessarily produced gradually. In regard to chloroform, however, even a fatal dose occupied but a very small space in the form of vapor, and unless great care was taken to have it largely diluted with air, it might act with dangerous rapidity, and the point of safety might easily be overstepped. The quantity of amylene which required to be inhaled, occupied, in the form of vapor, a volume intermediate between that of the vapor of chloroform and that of ether, and in all the ordinary methods of inhalation it must become mixed with a large portion of air. The relative advantages of amylene might be summed up as follows: In regard to its odor, it was more objectionable than chloroform, but much less so than sulphuric ether. In the amount which sufficed to induce insensibility, it was also intermediate between these two agents. In regard to its pungency, it had a great advantage over both ether and chloroform, being much less pungent than either of them; on this account the patient could always begin to inhale the amylene of full strength within half a minute, and the operation might generally be commenced within three minutes. It had an advantage in preventing pain, with a less deep stupor than was occasioned by the other agents, and in the ready waking and recovery of the patient, it had an advantage over chloroform, and a still greater advantage over ether. The almost entire absence of struggling and rigidity in the use of amylene is another advantage it possesses; and the greatest advantage of all, if it should continue to be met with, is the absence of sickness from its use.

Dr. Richardson had seen three cases in which amylene had been administered by Dr. Snow. He thought the stages of narcotism were not so well-marked in these cases as in those in which chloroform was administered. In the first case, the man became insensible to pain in three minutes and a half; in the second case, a child, in two minutes; and in the third case, a man, in one minute fifty seconds. The man's pulse was 134, and the respirations 60, in the minute. The most remarkable feature in this case was, the perfect quietude of the patient. Amylene, in its effects, was more allied to the common coal-gas. Mr. Nunneley had tried this agent, and would have persevered in its use had it not been so offensive in its odour, etc. Dr. Snow's patients had recovered from the effects of the amylene very rapidly. Neither of them appeared to be quite unconscious, though perfectly insensible to pain. He (Dr. Richardson) considered the simplicity of the compound—a hydro-carbonate—in favor of its employment. It had struck him that the extreme cold produced by the evaporation of amylene would render it a most useful means of producing local anæsthesia.—*Lancet,* Jan. 17, 1857.

At a subsequent meeting of the Society, Dr. Snow showed a specimen of amylene which had a less powerful and more agreeable odor than that which he showed to the Society on a former occasion. He said that the change had been produced by great care in its preparation on the part of Mr. Bullock, and that the chief obstacle to the use of this agent was in a great measure removed; and he expected that the odor would be still less, when the amylene could be procured in a state of more absolute purity. He had given the amylene in 69 operations, and in case of labor since he read the paper on Jan. 10th, making a total of 91 cases. The results confirmed what he had stated on the former occasions, as to certain advantages it possessed over chloroform in a number of instances. A little vomiting had occurred in six of the cases; this was much less than would be met with from chloroform, more especially as many of the patients had taken a meal just before the operation.—*Ibid,* March 6, 1857.

### METEOROLOGICAL NOTES.

[We publish below notes of Meteorological Observations, by Dr. J. M. Shaffer, of Fairfield, Iowa.

Fairfield has been selected by the Smithsonian Institute as a point for observations in this state, and Dr. Shaffer has been selected to take the observations and make up the record.

The value of such labor to the profession and to the community is now too well known and appreciated to need comment from us. Climactric influences, in their effects upon diseases, have been for a long time observed and studied by medical men; but accurate statistics have been wanting to a great extent; and as a consequence conclusions must have been more or less defective. Through the efforts of those connected with the Smithsonian Institute, Meteorological facts are being collected from every portion of our broad domain, and that these may be made of immeasurable value in the study of endemic and epidemic, and perhaps of general diseases, there is no room for question.

We hope to be able to secure from Dr. Shaffer a bi-monthly record of his observations for publication in the Journal. In the meanwhile we have to thank him for his politeness in sending us that which we now publish. Eds.]

In order that your readers may see the difference in the atmospheric conditions, in different years, even in the same latitude, we copy the table of 1855, as taken at the same points and with the same instruments:

WEATHER TABLE—1855,

| | Clear days | Cloudy days | Rain | Snow | Thunder | Highest temperature | Lowest temperature | Average temperature | Clear nights | Cloudy nights | Deaths |
|---|---|---|---|---|---|---|---|---|---|---|---|
| *January,* | 13 | 12 | 4 | 4 | | 71 | 8 | 21 | 13 | 18 | 3 |
| *February,* | 12 | 11 | 1 | 4 | | 52 | 5 | 23 | 16 | 12 | 1 |
| *March,* | 15 | 12 | 4 | 5 | 1 | 64 | 4 | 35 | 16 | 15 | 1 |
| *April,* | 13 | 14 | 7 | | 7 | 93 | 24 | 61 | 17 | 13 | |
| *May,* | 16 | 11 | 5 | | 4 | 96 | 31 | 68 | 19 | 12 | 4 |
| *Juue,* | 15 | 13 | 5 | | 3 | 93 | 45 | 69 | 20 | 10 | 1 |
| *July,* | 13 | 14 | 10 | | 12 | 99 | 59 | 75 | 20 | 11 | 8 |
| *August,* | 15 | 10 | 9 | | 6 | 98 | 49 | 62 | 23 | 8 | 18 |
| *September,* | 5 | 18 | 7 | | 4 | 98 | 38 | 70 | 15 | 15 | 3 |
| *October,* | 20 | 8 | 3 | 3 | | 80 | 22 | 52 | 25 | 6 | 1 |
| *November,* | 18 | 9 | 5 | | 1 | 90 | 12 | 53 | 12 | 12 | 3 |
| *December.* | 15 | 11 | 3 | 5 | | 56 | 16 | 22 | 15 | 16 | 1 |

TABLE OF 1856.

| | Clear days | Cloudy days | Rain | Snow | Thunder | Highest temperature | Lowest temperature | Average temperature | Clear nights | Cloudy nights | Deaths |
|---|---|---|---|---|---|---|---|---|---|---|---|
| *January,* | 11 | 12 | | 8 | | 26 | 26 | 12 | 16 | 15 | 2 |
| *February,* | 14 | 9 | 1 | 5 | | 46 | 22 | 20 | 16 | 13 | 2 |
| *March,* | 15 | 13 | | 3 | | 64 | 5 | 33 | 20 | | 3 |
| *April,* | 15 | 10 | 5 | | 3 | 84 | 30 | 55 | 23 | 7 | 2 |
| *May,* | 12 | 9 | 10 | | 3 | 93 | 41 | 64 | 22 | 9 | 2 |
| *June,* | 19 | 9 | 2 | | 1 | 100 | 50 | 74 | 19 | 11 | |
| *July,* | 18 | 6 | 9 | | 8 | 97 | 62 | 89 | 20 | 11 | |
| *August,* | 19 | 9 | 3 | | 3 | 88 | 46 | 73 | 26 | 5 | 1 |
| *September,* | 19 | 7 | 4 | | 2 | 93 | 32 | 63 | 23 | 7 | 2 |
| *October,* | 14 | 8 | 9 | | 3 | 87 | 22 | 57 | 22 | 9 | 5 |
| *November,* | 8 | 14 | 5 | 3 | | 58 | 5 | 36 | 8 | 22 | |
| *December.* | 13 | 6 | 6 | 6 | 1 | 45 | 8 | 19 | 13 | 18 | 4 |

COMPARATIVE TABLE.

| | Clear days | Cloudy days | Rain | Snow | Thunder and Lightning | Highest temperature | Lowest temperature | Clear nights | Cloudy nights | Deaths |
|---|---|---|---|---|---|---|---|---|---|---|
| 1855. | 170 | 143 | 63 | 21 | 38 | 99 | 16 | 217 | 148 | 44 |
| 1856. | 177 | 112 | 54 | 25 | 24 | 100 | 26 | 228 | 138 | 23 |

COLDEST DAYS OF 1856.

| | | | | | | | |
|---|---|---|---|---|---|---|---|
| January | 3d, | 9 | degrees, | February | 2d, | 8 | degrees. |
| " | 7th, | 8 | " | " | 3d, | 13 | " |
| " | 8th | 17 | " | " | 4th, | 6 | " |
| " | 9th | 11 | " | Dec, | 22d, | 1 | " |

In the above days the average of the entire day is taken. The coldest range was 26 degrees, on Wednesday morning, January 9th.

HOTTEST DAYS OF 1856.

| | | | | | |
|---|---|---|---|---|---|
| May 23d, | 80 degrees | | July 6th, | 78 degrees, | |
| " 24th, | 81 | " | " 9th, | 79 | " |
| June 3d, | 82 | " | " 23d, | 82 | " |
| " 23d, | 89 | " | " 24th, | 82 | " |
| " 24th, | 82 | " | Sept. 9th, | 83 | " |

Here, too, the general average is taken. At noon, on several days, the range was 98 deg. The highest degree of temperature was 100, at noon of June 23d.

SPECIAL NOTES.

The winter of '55--6 is said to have been unequalled in its intensity of cold weather for fifteen years. The coldest continuous days were from January 3d to 10th inclusive. It may be of interest to state the entire range at that period.

| 1856. | | | | | 1857. | | | | |
|---|---|---|---|---|---|---|---|---|---|
| January | Morning | Noon | Sunset | 10 P. M. | January | Morning | Noon | Sunset | 10 P. M. |
| 3d | 16 | 6 | 5 | 10 | 17th | 14 | 6 | 12 | 18 |
| 4th | 3 | 14 | 14 | 12 | 18th | 24 | 12 | | 15 |
| 5th | 8 | 13 | 4 | 5 | 19th | 8 | 12 | 20 | 22 |
| 6th | 12 | 20 | 19 | 8 | 20th | 18 | 12 | 7 | |
| 7th | 16 | 4 | 4 | 6 | 21st | 8 | 3 | 8 | 18 |
| 8th | 28 | 11 | 17 | 21 | 22d | 15 | 10 | 3 | 5 |
| 9th | 25 | | 6 | 12 | 23d | 3 | 8 | 5 | 5 |
| 10th | 15 | 11 | 8 | 5 | 24th | 5 | 6 | 5 | 5 |

Anticipating somewhat, we have annexed a table of the coldest consecutive days of this year, occurring two weeks later than in 1856. By a calculation, the average for the eight days of 1856 is not quite two degrees below zero; while the average the present year is 1½ degrees below zero—showing that the present winter is nearly as cold as the last. Saturday night, January 26, occurred a furious snow storm—drifted in large masses from the northeast.

February 15th, after a succession of cold days, became quite warm. Snow melts away with singular rapidity, high top boots in demand, lady pedestrians scarce. From this date to the 22d it was quite moderate. Range about 34 degrees. On the 22d it began to rain at 1 P. M., and continued steadily up to 6, then turned to snow, Frost out of the ground to the depth of three inches; snow mostly disappeared, except in the largest drifts; water standing in large pools on certain lots, and drains running full. This was the first rain since Dec. 8th, a period of seventy-six days. From the 22d to the 29th the roads were very muddy.

April 4. Roads begin to improve. After about three weeks of mud and frozen ground, and as early as April 15th, the public roads were in excellent condition.

April 26th, was the only hail storm of note during the year. This storm was not general, occurring in distinct strips in several parts ot the country. No material damage done. For several days previous, an unusually strong west wind prevailed.

The first two weeks in May were marked by a great deal of rain, with numerous thunder storms. Rains wash out the bridges, and otherwise make the roads very bad. The latter part of the month was very mild and agreeable.

In June, with a great deficiency of rain, we had a profusion of circuses and shows, which were rather largely patronised. June 25th is the following note : A fine shower at 6 A. M.; also a rain at noon quite sufficient to lay the dust, and moisten the ground a couple of inches, the first rain to produce such an effect since May 14th. Much of the corn planted failed to come up; re-planted, did not succeed; at this writing is about advanced enough for a first ploughing. Irish potatoes promise half a crop; sweet potatoes commencing to vine; will be mostly a failure; garden vegetables well nigh burnt up. Many young trees planted out last year, began ta grow, but were parched in the drought. Timothy light; clover, abundant crop; peach trees almost universally killed during the winter; apples fall off, and in some places the weevil has ruined the crop; cherries abundant; wild plums an extraordinary crop; grapes winter killed. A large number of locust trees, the growth of eight or ten years, failed to grow, or maintained a sickly growth for a few weeks and died. (Compare this note with Dec. 1st.)

July was ushered in by several heavy storms. This month compares with July of 1855, in being the hottest month of the year. Mornings usually calm, succeeded by a strong south-west wind, which carries with it large, fleecy clouds during the day, subsides at night and becomes clear. On the 2d, before morning, was a severe storm; cloudy all day; clear at 5 P. M.; clouds came up from the west at 8 P. M., with vivid lightnings; at 12 commenced to rain with a fierce wind; continued for three hours, being the heaviest rain for three years. Jas. Herron found his horse in the meadow at six o'clock in the morning, with one leg completely shattered and torn up, the effects of a stroke of lightning.

August was a very pleasant month. A considerable depression of temperature below last month ; most of the days clear, very little rain, and not one sudden change.

September does not present any remarkable peculiarity. We notice that the ordinary house fly becomes troublesome on the 15th; previously scarcely one was to be seen in all our borders. 20th was the first frost at all noticeable.

October did not sustain the reputation of this climate for beautiful fall weather, on account of unusual rains, fogs, and great variableness. Winds generally west.

November. On Monday 3d, the first snow fell, at 5 P. M.; snowed very fast for two hours. This month was disagreeable from the same causes as noted in October.

December commenced with some heavy rains. These rains, coupled with snow, covered the country with water, and filled up the sloughs to an unusual extent. Upon this succeeded the cold weather of the 14th and 22d. The roads were almost impassable from the perfect glare of ice. Turn where you would, and the ice lay in masses all about you. 27th, occasional flashes of lightning in the north; rained all the previous night, and most of the day. This rain lay upon the ground, was frozen up, and rendered traveling dangerous and tedious. The "oldest settler" has no memory of such icy times. December 1st is this note : Corn sells readily at 25 cents per bushel. The average yield was pretty good; say sixty-five bushels per acre. Irish potatoes at 50 cents, double last year's price; find a general scarcity, from partial failure of the crop. Sweet potatoes sold at $1,25, a larger price than at any previous season. In many places the crop was almost a failure. Garden vegetables grew very finely after the June rains, and gave a good crop. Turnips unusually fine To show how variable our climate is, we append a table commencing December 20th, including 27th :

| | Morn. | Noon. | Sunset | 9 P. M. | NOTES. |
|---|---|---|---|---|---|
| 20th | | 10 | 12 | 10 | Clear ; wind north west. [wind north west. |
| 21st | 10 | 32 | 24 | | Clear, 4 h ; cloudy ; snows. |
| 22d | 8 | 5 | 2 | 2 | Clear ; wind west. |
| 23d | 2 | 12 | 17 | 15 | Cloudy ; wind south-east. |
| 24th | 13 | 33 | 28 | 20 | Clear ; wind south. |
| 25th | 18 | 32 | 30 | 22 | Cloudy ; wind south-east. |
| 26th | 24 | 32 | 33 | 30 | Cloudy ; wind east. |
| 27th | 32 | 34 | 43 | 35 | Rains ; wind west. |

The differences here are sufficiently obvious ; and this table is not an extraordinary one. Changes are often more marked and sudden than any noted here.

Many important deductions and inferences could be made from the foregoing observations. We will not enlarge here, but would especially call your attention to the deaths of 1855 and 1856. Notice the difference in the number of deaths, the time of their occurrence, the nature of the diseases, and the attending atmospheric phenomena. The following comparative tables will assist in these reflections :

| 1855: Whole Number. | 44 | 1856: Whole Number, | 23 |
|---|---|---|---|
| Under two years, | 22 | Under two years, | 9 |
| " ten years, | 6 | " ten years, | 5 |
| Between ten and twenty years, | 0 | Between ten and twenty years, | 3 |
| Betw. twenty and fifty years, | 13 | Betw. twenty and fifty years, | 3 |
| Over fifty years, | 3 | Over fifty years, | 3 |
| PRINCIPAL DISEASES. | | | |
| Dysentery, | 24 | Dysentery, | 0 |
| Lung Fever, | 3 | Lung Fever, | 2 |
| Child Bed, | 2 | Child Bed, | 1 |
| Cholera, | 3 | Cholera, | 0 |
| Consumption, | 1 | Consumption, | 4 |
| Scarlet Fever, | 0 | Scarlet Fever, | 3 |
| Croup, | 1 | Croup, | 1 |
| Congestive Fever, | 1 | Congestive Fever, | 2 |
| Diarrhœa, | 0 | Diarrhœa. | 3 |

The other deaths in 1856 were from Convulsions, Paralysis, Dyspepsia, Inflammation of the Bowels, Cynaosis, Debility and Quinzy, each one.

No epidemic visited us during the entire year. The health was considered remarkably good, both in the village and in the surrounding country. Early in December a singular affection of the throat, involving the tonsils and also the glands about the ear and the angle of the jaw, commenced. It presented some remarkable features. A complete history of this disease has been recorded, with a view to its publication in another place. It will more properly belong to a review of 1857.

From the period of the entire disappearance of frost from the ground up to its first occurrence in the fall, was a period of 145 days. This gives us but a very short summer season, leaving 221 days not fitted to the growth of plants and trees. We submit this paper to your readers without comment, hoping it will be as favorably received as other efforts of a like kind, at other times.

J. M. Shaffer.

*Fairfield, Feb.* 18, 1857.

### Sickness and Death of Dr. Kane.

[Communicated to the Boston Society for Medical Improvement, by F. S. Ainsworth, M. D.]

The death of the late Dr. E. K. Kane, which took place in Havana on the 16th of February, though not unexpected, has still filled the minds of all who knew him with deep regret, that a career so brilliantly commenced and so faithfully followed should be prematurely terminated. It was the fortune of the writer to attend him, in consultation with his regular physician, (Dr. La Riverend,) during the last part of the sickness which terminated his life. A few particulars of his case, gathered in that short period, will, it is believed, derive some interest from their connection with one so justly celebrated.

Dr. Kane inherited a decided predisposition to rheumatic affection, and had from early life been subject to attacks of articular rheumatism. He suffered very severely from the disease after his return from the first Arctic expedition. The heart also had become involved, and he was thought to have a considerable degree of hypertropy, together with thickening of the valves. So severely was he afflicted with articular rheumatism while preparing for the last cruise in search of Sir John Franklin, that it was often necessary to apply frictions to the joints an hour before rising in the morning, in order to enable him to ride to the navy yard, where the "Advance" was fitting out.

Very soon after getting into the high latitudes, however, these difficulties subsided—a result which would hardly have been anticipated, but which he had observed in his own case on his previous voyage. What his sufferings and exposures were during his Arctic expedition, is well known; but it is proper to state that they were much more severe, and their effect upon his constitution more disastrous, than would be supposed from the few allusions made to his own case in the published account of the expedition.

On his return, his previous rheumatic and cardiac troubles had become complicated with scurvy; though very much exhausted and worn out by the hardships he had undergone, he allowed himself no time for repose, but labored incessantly in preparing the account of his expedition for publication. This fatigue, together with the great change in climate and habits, brought on a severe relapse of his constitutional disease, aggravated by the newly acquired scorbutic taint. He received little or no benefit from the treatment of his disease while in this country, and was advised to try a change of climate; accordingly, after the publication of his book, he sailed for England. Here his health became much better, all his symptoms were much improved, and he considered himself nearly restored to health. As, however, there still remained some traces of scurvy about him, his physicians advised him to spend the winter in the West Indies, for the benefit of the climate and fruits.

Since his return from the north, there was a somewhvt remarkable change in his ability to bear the motion of the ship. He had become unusually sensitive to sea sickness, which was brought on by even a slight rolling of the vessel. The voyage from London to St. Thomas was, however, well supported. While there, his health continued to improve, and at the end of six weeks he sailed for Havana. The ship in which he took passage was overtaken by a severe storm; he was much affected by the motion of the vessel, and in the effort and strain of vomiting ruptured a blood vessel in the brain. Entire insensibility followed, and continued for several days after his arrival in Havana. A partial recovery took place after a few days, but the right side was found to be completely paralyzed.

During the months of December and January and until the 10th of February, he slowly rallied from this attack, and was able to walk a little about his room and to drive out. He recovered the use of the right hand and wrist to a great degree, and shortly before the second attack was able to rotate the fore-arm. His mind was perfectly clear, although there was some loss of control over the memory. When he endeavored to recall any circumstance which had transpired, several others, more or less connected with it, were remembered, from which he was unable to isolate the particular fact desired. Of this difficulty he was himself perfectly conscious.

On the 10th of February, at the morning visit, he appeared more cheerful than usual, and conversed a good deal with those about him. About 11 o'clock, however, he was suddenly seized with a severe attack of apoplexy, which deprived him entirely of consciousness. There was considerable spasmodic action of the muscles, which simulated in some degree a fit of epilepsy. These symptoms soon subsided leaving him with almost complete paralysis of the entire body. The iris responded to light, and the muscles of the phalynx acted when stimulated by fluid introduced into the mouth. The pulse was feeble and varied from 120 to 140 beats. The skin was moist and cool. He remained very much in this state until his death, which took place on the fifth day after the seizure. In this interval, however, he seemed to have recovered some degree of consciousness, and several times signified assent to a question by turning his eyes towards the speaker.

There was some motion of the lips when a spoon was placed in the mouth, and once or twice he was able to make sensible pressure with his right hand. There was no indication of suffering during his last hours, and he died apparently from simple exhaustion.

The tenacity of life in this case was quite remarkable. A constitution broken by chronic disease of many years' standing ; a series of hardships and exposures almost unheard of, with all the depressing addition of care and responsibility, followed by an affection which for some months threatened his life ; add to all these an attack of apoplexy, paralyzing entirely the right side, and in two months after a relapse affecting the whole body, and one can hardly conceive how life could have been sustained for so long a period as five days after the last shock.

The treatment in this case was quite simple. On account of his previous illness and the scorbutic taint in his system, it was thought unsafe to resort to the active measures usually pursued in such cases. After the first attack, small doses of ext. nux vomica with quinine were administered. These were suspended after a time, through fear of increasing the cardiac disease, and a high tonic and antiscorbutic course was followed. After the second attack, a few leeches were applied, together with cold applications to the head.

[*Boston Med. and Surg. Journal.*

